# *NDUFAF5* variants manifest phenotypically heterogeneously

**DOI:** 10.1016/j.ymgmr.2018.11.005

**Published:** 2018-12-11

**Authors:** Josef Finsterer

**Affiliations:** Krankenanstalt Rudolfstiftung, Messerli Institute, Veterinary University of Vienna, Vienna, Austria

**Keywords:** Mitochondrial, Leigh syndrome, Stroke-like episode, Multisystem disease, Respiratory chain

Letter to the Editor

With interest we read the article by Simon et al. about 4 patients with complex-I deficiency due to mutations in the *NDUFAF5* gene [1]. The phenotype and the outcome of these 4 patients varied considerably [1]. We have the following comments and concerns.

The main shortcoming of this case series is that no prospective studies for multisystem involvement had been carried out. Multisystem disease in mitochondrial disorders (MIDs) may be subclinical or mild and thus frequently overlooked, why these patients need to be systematically investigated for multiorgan affection. Additionally, no comprehensible explanation for the marked phenotypic variability was provided.

MRI in patient-4 revealed DWI hyperintense serpiginous lesions [1]. To clarify if these lesions represent a cytotoxic or vasogenic edema it would be helpful to know the results of the ADC maps. Hypointensity of the ADC maps would suggest cytotoxic edema and thus ischemia, whereas hyperintense ADC maps would suggest vasogenic edema and thus a stroke-like lesion, which would require treatment with NO-precursors, antiepileptic drugs (AEDs), antioxidants, and steroids [2].

In patient-3 arterial hypertension was attributed to administration of ACTH (Table 1) [1]. However, patient-1 had arterial hypertension in the absence of a plausible trigger. Since arterial hypertension has been repeatedly reported as a primary manifestation of mitochondrial disorders (MIDs) [3], it is conceivable that arterial hypertension in patient-1 as well as patient-3 was a primary phenotypic manifestation of the *NDUFAF5* mutation.

**Table 1:** Findings in the 4 reported patients.

Patient	1	2	3	4
Ethnicity	Taiwanese	Taiwanese	Caucasian	Ashkenazi
Age	10 m	7 y	4 m	12 m
Sex	Female	Female	Male	Male
Onset	6 m	27 m	1 m	Birth
Onset manifestation	Dysphagia	Strabism	Reflux	Develop. delay
Phenotype	Head drop	Oligohydramnion	Irritability	Torticollis
	Nystagmus	Ptosis	Insomnia	Nystagmus
	Blepharospasm	Vomiting	Seizures	Head bobbing
	Vomiting	Optic atrophy	Truncal hypotonia	Hypotonia
	Hypotonia	Fatigue	Limb spasticity	Dysmorphism
	Weight loss	Muscle Weakness	Cortical blindness	Dysphagia
	SIADH	Headache	Dysphagia	Vomiting
	Hypertension	Tremor		
		Contractures		
		Spasticity		
		Hypertension		
		Seizures		
		Pruritus		
		GI dysmotility		
Hyponatriemia	Yes	Yes	No	No
Lactic acidosis serum	Yes	No	Yes	Yes
Lactic acidosis CSF	nd	No	Yes	Yes
Amino acids serum	nd	Normal	Abnormal	nd
Abnormal UOAs	Yes	Yes	Yes	No
MRI lesions	Thalamus	Medulla	Thalamus	Thalamus
	Midbrain	Upper cervical cord	Midbrain	DWI
	CC dysgeneis			
Muscle biopsy	nd	Fat droplets	RRF	nd
Biochemistry	nd	Normal	C-I defect	nd
Gene	NDUFAF5	NDUFAF5	NDUFAF5	NDUFAF5
Mutations	c.155A > C	p.Met279Arg	c.327G > C	c.327G > C
	c.836 T > G			c.749G > T
Treatment	Thiamine	Thiamine	ACTH	Biotin
	Coenzyme-Q10	Coenzyme-Q	Hydralazine	Pantotheniacid
	l-carnitine	Riboflavin	Amlodipine	Thiamine
	Propranolol	L-carnitien	Enalapril	l-carintine
	Hydrochlorothiazide	Labetalol	Vigabatrin	Fundoplication
		Propranolol		
		Captopril		
		Gabapentin		
		Lisinopril		
		Enalapril		
Outcome	Death at 25 m	Alive at 19 y	Death at 6 m	Death at 17 m

SIADH: syndrome of inappropriate atidiuretic hormone secretion, CSF: cerebrospinal fluid, UOAs: urine oranic acids, CC: corpus callosum, GI: gastrointestinal, RRF: ragged-red fibers, ACTH: adreno-corticotropic hormone, nd: not done.

Both Taiwanese patients had severe hyponatremia (Table 1) [1]. We should be informed if this was due to primary or secondary hypoaldosteronism, or due to pituitary insufficiency, occasionally occurring as endocrine manifestations of a MID [4].

Overall, this case study could be more meaningful if clinical variability and MRI-findings in patient-3 would have been more extensively discussed and if all patients would have been systematically investigated for multisystem involvement.

## Conflict of interest

There are no conflicts of interest.

## Funding

No funding was received.

## References

[1] Simon M.T., Eftekharian S.S., Stover A.E., Osborne A.F., Braffman B.H., Chang R.C., Wang R.Y., Steenari M.R., Tang S., Hwu P.W., Taft R.J., Benke P.J., Abdenur J.E. (Nov 08, 2018). Novel mutations in the mitochondrial complex I assembly gene NDUFAF5 reveal heterogeneous phenotypes. Mol Genet Metab..

[2] Finsterer J. (2018). Stroke-like episodes in coenzyme-Q deficiency may respond to NO-precursors and non-mitochondrion-toxic antiepileptic drugs. Mol Genet Metab Rep.

[3] Xu Y., Chen X., Huang H., Liu W. (2017). The mitochondrial tRNA(Ala) T5655C mutation may modulate the phenotypic expression of tRNA(Met) and tRNA(Gln) A4401G mutation in a Han Chinese family with essential hypertension. Int. Heart J..

[4] Finsterer J., Zarrouk-Mahjoub S. (2018). Letter to the Editor: Endocrine compromise in mitochondrial disorders. J Endocr Soc.

